# Epoetin Alfa: A Cause of Coronary Artery Thrombosis

**DOI:** 10.1155/2017/9475180

**Published:** 2017-10-01

**Authors:** Muhammad Umer Siddiqui, Yelena Galumyan, James Klein, Zunaira Naeem, Aron Schwarcz

**Affiliations:** ^1^Lakeview Medical Center, Marshfield Clinic, Rice Lake, WI, USA; ^2^Englewood Hospital and Medical Center, Englewood, NJ, USA; ^3^DOW Medical College, Karachi, Pakistan

## Abstract

**Introduction:**

Epoetin alfa is an erythrocyte-stimulating factor. We here present a case of an anemic patient, who was given epogen before a coronary artery bypass (CABG) surgery and developed periprocedural myocardial infarction. To our knowledge, there has been no previous case reported of epogen causing myocardial infarction.

**Case Presentation:**

66-year-old female presented with substernal chest pain. EKG showed ST segment elevations in aVf and L-III. Patient underwent left heart catheterization (LHC) and had triple vessel disease. A bare metal stent was placed in RCA. Patient continued to have symptoms after discharge and presented to the ED again with these complaints. She underwent coronary artery bypass surgery (CABG). Before the procedure, patient's hemoglobin was 11.1 grams/deciliter and was given epogen to raise hemoglobin level. Postoperative day (POD) #1 patient had ST elevations in inferior and anterolateral leads. She was diagnosed with periprocedural myocardial infarction. Patient underwent repeat LHC, which identified obstruction of saphenous vein graft (SVG). Hypercoagulable workup was negative for any disease and the patient was diagnosed with epogen induced early graft occlusion.

**Conclusion:**

Epogen administration can cause myocardial infarction and coronary artery thrombosis. We suggest that physicians use epogen very cautiously, especially in population who has underlying coronary artery disease.

## 1. Introduction

Epoetin alfa, also known as epogen, is a recombinant human erythropoietin and is used commonly in anemic population with chronic kidney diseases to increase their production of red blood cells. However, it is associated with some serious side effects like thromboembolism and acute coronary syndromes [1–3]. Herein, we discuss a challenging case of an anemic Jehovah's Witness who required a coronary artery bypass surgery (CABG) and was prescribed epoetin alfa prior to CABG surgery. 

## 2. Presentation

**Figure 1:**
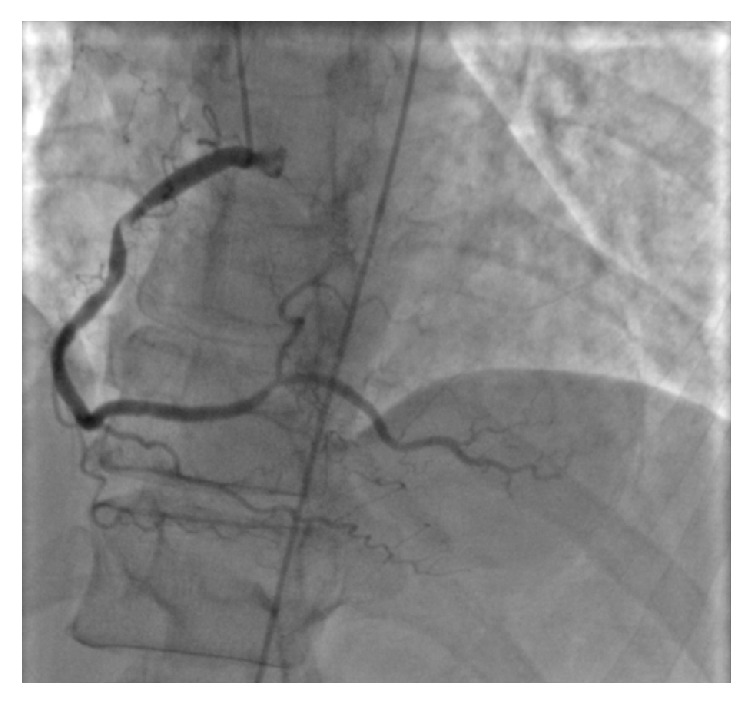
Left heart catheterization identifying stenosis of proximal right coronary artery.

A 66-year-old woman with history of hyperlipidemia and hypothyroidism initially presented with pressure-like substernal chest pain radiating to the jaw. Her EKG showed ST segment elevations in augmented vector foot (avf) and limb lead III (L-III) with increased serum cardiac enzymes. She was emergently taken to the catheterization lab and was found to have a 95% stenosis in the proximal right coronary artery (RCA) (Figure 1), a 60% stenosis in the diagonal 1 branch, and a 75% stenosis in middle left anterior descending artery (mLAD) lesion. The RCA was thought to be the culprit lesion and a bare metal stent (BMS) was placed. However, after the discharge, she continued to have fatigue and lack of energy over the following few weeks. She was also having shortness of breath and chest discomfort when going up 1 flight of stairs or when walking short distances. For these reasons, she came back to the emergency department and was reevaluated. Since she had a known history of triple vessel coronary artery disease on a recent cardiac catheterization, coronary artery bypass surgery (CABG) was recommended. Prior to the procedure, her hemoglobin was 11.1 grams/deciliter. As she was Jehovah's Witness and did not accept any blood products, she was given epoetin alfa 20,000 units subcutaneous daily two days before surgery, on the day of surgery, and two days after surgery to raise her hemoglobin level. This step was taken to maximize her preoperative hemoglobin level to balance the expected blood loss during surgery. She underwent a triple vessel bypass surgery with left internal mammary artery (LIMA) to left anterior descending artery (LAD), saphenous vein graft (SVG) to the diagonal number 1, and SVG to right posterior descending artery (RPDA). Her left ventricular ejection fraction was 50% immediately pre- and postoperatively.

**Figure 2:**
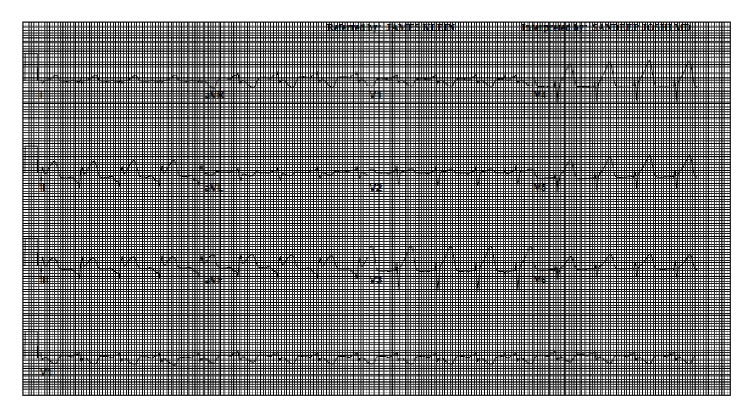
Post-CABG EKG showing ST segment elevations in L-II, L-III, aVF, and V3–V6.

On postoperative day (POD) 1, she was noted have to have new EKG changes, including ST segment elevations in the inferior and anterolateral leads (Figure 2). She was however symptom free and these were thought to be postoperative changes. Her medications were optimized and she was closely observed in the cardiac care unit (CCU). Her cardiac enzymes peaked on POD#3 and then started trending down. However, on POD#7 she became lethargic and hypotensive. Here mental status and blood pressure improved after IV fluid boluses. On POD#8, she complained of shortness of breath and orthopnea. Chest X-ray revealed new bilateral pleural effusion and chest tubes were reinserted.

## 3. Assessment

For the reasons stated above, she underwent a repeat echocardiogram (echo), which showed a significant drop in the ejection fraction to 15%. It also identified new akinesis of mid to distal anteroseptal, inferior, and apical walls consistent with the EKG changes. As she had new areas of infarct on the echo, a viability study was performed prior to undergoing further workup. A nuclear viability study showed mild viability of the apex and septum and full viability of the inferior wall.

**Figure 3:**
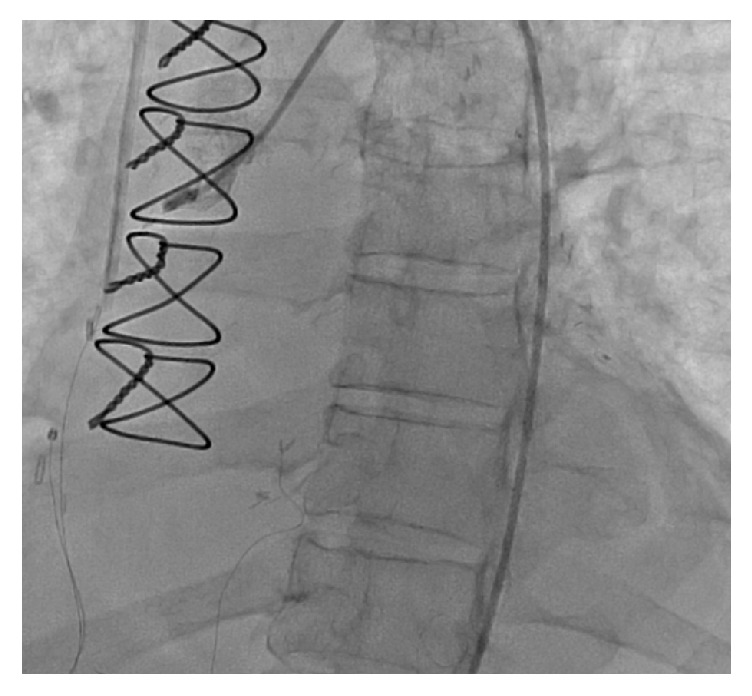
Left heart catheterization showing occlusion of SVG graft to RPDA.

**Figure 4:**
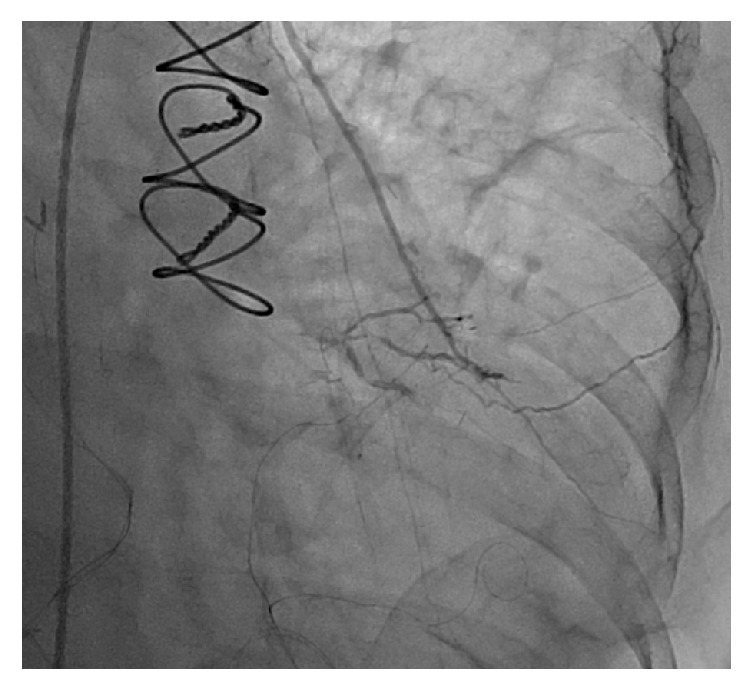
Left heart catheterization showing diseased LAD distal to LIMA insertion.

**Figure 5:**
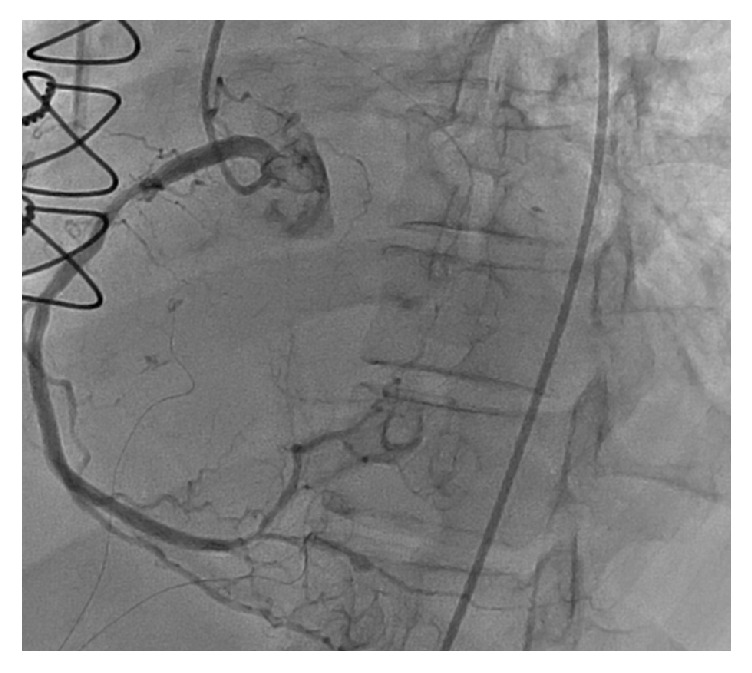
Left heart catheterization identifying worsening disease of RPDA after CABG.

The decision was made to perform a repeat left heart catheterization. Repeat catheterization showed occlusion of SVG graft to RPDA (Figure 3), thrombosis of the LAD distal to the LIMA insertion (Figure 4), and thrombosis of the RPDA (Figure 5). There were no adequate targets for revascularization and it was decided to treat her medically. Her medications were optimized and patient was discharged few weeks later in a well-compensated state to subacute rehabilitation with outpatient follow-up in 2-3 weeks.

## 4. Diagnosis

Patient had fair distal vessels and procedure was uneventful. After multidisciplinary discussion, a full hypercoagulable workup was initiated. The workup included Factor V Leiden mutation, prothrombin (factor II) mutation, protein C deficiency, protein S deficiency, and antiphospholipid antibody, which all came back negative. After the extensive workup and discussion with hematology-oncology, the early SVG graft occlusion was attributed to the administration of epoetin alfa before the CABG surgery.

To our knowledge, there has been no other published case report of epoetin alfa causing myocardial infarction reported before.

## 5. Discussion

This is a challenging case in terms of diagnosis and management. Our patient was a Jehovah's Witness and did not accept blood transfusion. She was anemic on presentation and required a high-risk surgery. Therefore, after several multidisciplinary meetings, it was decided that she should receive epoetin alfa to raise her hemoglobin before surgery. The data showing epoetin alfa causing cardiovascular thrombosis is very limited. Below we will discuss the current data regarding epoetin alfa's side effects.

One of the side effects of epoetin alfa is iatrogenic polycythemia causing ischemia secondary to thromboembolism. A meta-analysis review was performed on several trials evaluating the use of erythrocyte-stimulating agents for the treatment of anemia in the oncology setting. 51 trials with 13,611 patients were analyzed and showed a 1.57-fold increased risk of venous thromboembolism and increased mortality associated with use of erythrocyte-stimulating agents [1]. This trial, however, did not evaluate if there is an increased risk of coronary artery thrombosis associated with epoetin alfa.

Our patient had myocardial infarction secondary to epoetin alfa administration and an association between epoetin alfa and cardiovascular harm was initially described in the Normal Hematocrit Study [2]. This study analyzed 1233 patients with clinical evidence of congestive heart failure or ischemic heart disease that were undergoing dialysis. One group received increasing doses of epoetin alfa to reach and maintain a normal hematocrit value of 42 ± 3% and the other group continued epoetin alfa therapy to maintain a hematocrit value of 30 ± 3%. They found that 33% of the patients in the normal hematocrit group died or had a nonfatal myocardial infarction, as compared to 2% of those in the low hematocrit group (risk ratio 1.3; CI 0.9–1.9).

Other trials like the Correction of Hemoglobin and Outcomes in Renal Insufficiency (CHOIR) trial also found increase risk of death, myocardial infarction, and congestive heart failure in patients with higher hemoglobin (13.5 grams per deciliter) achieved through epoetin alfa as compared to patients with low hemoglobin (11.3 grams per deciliter) (hazard ratio 1.34; CI 1.03–1.74). The trial found that there was a nonsignificant trend towards increased myocardial infarction in patients with high hemoglobin as compared to low hemoglobin (hazard ratio 0.91, *p* value 0.78) and also a significant increase in hospitalization due to cardiovascular causes in the high hemoglobin group as compared to the low hemoglobin (hazard ratio 1.23; CI 1.01–1.48) [3].

Another randomized controlled trial performed on 1,460 critically ill anemic patients with hemoglobin less than 12 grams/deciliter found that when compared with placebo, epoetin alfa was associated with significant increase in the incidence of thrombotic events (hazard ratio. 1.41; 95% CI, 1.06–1.86). In this study, 21 patients suffered myocardial infarction out of which 15 were in the epoetin alfa group (*p* value: 0.08) [4]. During a review of the marketing application for darbepoetin alfa, an association was found between rate of increase in the hemoglobin level exceeding 1 gram per deciliter per 2-week period and the risk of cardiovascular and thromboembolic event [5]. Our patient had an increase in hemoglobin of 0.9 grams/deciliter within 2 days of the epoetin alfa initiation, which may have contributed in the development of thrombosis within her cardiovascular system.

The data on epoetin alfa predisposing to coronary artery thrombosis is limited and there are no concrete guidelines regarding the use of epoetin alfa. However, literature review has shown increase cardiovascular harm with its use. Raising hemoglobin in an anemic patient who does not accept transfusions before a high-risk surgery is beneficial [6]; however, in light of the current evidence, the goal should be to aim for an acceptable level rather than a normal level.

## 6. Conclusion

Our case report is unique, as a detailed literature search did not show any previous case report tackling this complicated issue. Our patient was anemic and in theory should have benefited with a higher hematocrit before surgery. However, epoetin alfa increases the risk of acute coronary syndrome as shown in this case report and in previous clinical trials. We recommend that epoetin alfa be used cautiously especially in population with heart disease and those receiving epoetin alfa should have ischemic evaluation performed if they develop chest pain or EKG changes.
